# 3D Printing of Cellulase-Laden Cellulose Nanofiber/Chitosan Hydrogel Composites: Towards Tissue Engineering Functional Biomaterials with Enzyme-Mediated Biodegradation

**DOI:** 10.3390/ma15176039

**Published:** 2022-09-01

**Authors:** Arnaud Kamdem Tamo, Tuan Anh Tran, Ingo Doench, Shaghayegh Jahangir, Aastha Lall, Laurent David, Carlos Peniche-Covas, Andreas Walther, Anayancy Osorio-Madrazo

**Affiliations:** 1 Laboratory for Bioinspired Materials BMBT, Institute of Microsystems Engineering IMTEK, University of Freiburg, 79110 Freiburg, Germany or; 2 Freiburg Center for Interactive Materials and Bioinspired Technologies FIT, University of Freiburg, 79110 Freiburg, Germany; 3 Freiburg Materials Research Center FMF, University of Freiburg, 79104 Freiburg, Germany; 4 Polymer Materials Engineering IMP CNRS UMR 5223, Université Lyon, Université Claude Bernard Lyon 1, Université Jean Monnet St Etienne, INSA de Lyon, CNRS, 69622 Villeurbanne, France; 5 Center of Biomaterials, Faculty of Chemistry, University of Havana, Havana 10400, Cuba; 6 ABMS Lab, Active, Adaptive and Autonomous Bioinspired Materials, Department of Chemistry, University of Mainz, 55128 Mainz, Germany

**Keywords:** 3D (bio)printing, enzyme immobilization, controlled release, cellulose nanofibers, chitosan, enzymatic biodegradation, casein, nanoparticles, cell culture, tissue engineering

## Abstract

The 3D printing of a multifunctional hydrogel biomaterial with bioactivity for tissue engineering, good mechanical properties and a biodegradability mediated by free and encapsulated cellulase was proposed. Bioinks of cellulase-laden and cellulose nanofiber filled chitosan viscous suspensions were used to 3D print enzymatic biodegradable and biocompatible cellulose nanofiber (CNF) reinforced chitosan (CHI) hydrogels. The study of the kinetics of CNF enzymatic degradation was studied in situ in fibroblast cell culture. To preserve enzyme stability as well as to guarantee its sustained release, the cellulase was preliminarily encapsulated in chitosan–caseinate nanoparticles, which were further incorporated in the CNF/CHI viscous suspension before the 3D printing of the ink. The incorporation of the enzyme within the CHI/CNF hydrogel contributed to control the decrease of the CNF mechanical reinforcement in the long term while keeping the cell growth-promoting property of chitosan. The hydrolysis kinetics of cellulose in the 3D printed scaffolds showed a slow but sustained degradation of the CNFs with enzyme, with approximately 65% and 55% relative activities still obtained after 14 days of incubation for the encapsulated and free enzyme, respectively. The 3D printed composite hydrogels showed excellent cytocompatibility supporting fibroblast cell attachment, proliferation and growth. Ultimately, the concomitant cell growth and biodegradation of CNFs within the 3D printed CHI/CNF scaffolds highlights the remarkable potential of CHI/CNF composites in the design of tissue models for the development of 3D constructs with tailored in vitro/in vivo degradability for biomedical applications.

## 1. Introduction

Tissue engineering is currently a booming field of research that focuses on the regeneration of damaged or missing tissues and organs [1,2,3]. This field of biomedical engineering ultimately uses a combination of cells, suitable biochemical factors and biomaterials, serving as support for cell growth and proliferation in vitro or during the formation of new tissues in vivo [4,5,6]. Three-dimensional scaffolds suitable for tissue regeneration should have or develop a porous microstructure, allow cell colonization and present suitable mechanical properties [7]. To design 3D hydrogel constructs, 3D printing using additive manufacturing technologies and appropriate inks commonly, prepared with polymer compounds, is increasingly used [8,9]. Three-dimensional printing offers the possibility of creating complex bioinspired constructs with target microstructure in which the cells can be precisely seeded at high densities in the pores of scaffolds and eventually ‘encapsulated’ in the biomaterial [10,11,12]. Many biomaterials have been used in the field of tissue engineering to attempt to regenerate a variety of tissues in vitro or in vivo. In vitro cell culture is usually performed (e.g., in cell medium) for tissue reconstruction. However, although these models often provide an adequate in vitro environment for cell proliferation, two-dimensional (2D) in vitro culture remains somehow limited because it does not take into account the physiological interactions to which cells are subjected in vivo in the 3D extracellular matrix to perform their regular functions. Moreover, these usual in vitro cell culture platforms do not faithfully mimic the physiological context and often suffer from imprecise spatial and temporal control of extracellular signals. Over the years, new concepts have evolved, such as porous scaffold architectures, capable of delivering biological and biochemical signals to the cells [13], allowing cells to appropriately interact with their extracellular environment in order to proliferate, differentiate and rebuild a new 3D tissue network, mimicking the native tissue. This concept typically uses biomaterials or biocomposites with enhanced physicochemical, biological properties, and eventually, biodegradability, to create a bioresponsive and penetrable scaffold that facilitates tissue regeneration in vivo.

Regardless of the type of tissue to be regenerated, a number of key issues must be considered in order to design biocompatible and biodegradable hydrogel scaffolds exhibiting the suitable porosity to ensure three-dimensional cell infiltration and proliferation in contact with a matrix with the adequate mechanical properties as well as diffusion of nutrients to the cells within the constructs [14,15]. The nature, composition and properties of the biomaterials used to prepare the inks as well as the cell types are, among others, essential aspects having an impact on the biofabrication process, physico-chemical features and properties of the resulting 3D scaffolds [16,17]. In this context, natural polymers are generally more attractive than synthetic polymers due to their biocompatibility, possible biodegradability and bioactivity and ability to mimic the properties of the natural extracellular matrix [18,19,20,21]. Natural hydrogels made from polymers, such as alginate [22,23,24], gelatin [25,26], cellulose [27,28] and collagen [29,30,31], are the most commonly studied materials for the development of 3D constructs by micro-extrusion. However, to improve their mechanical properties, these polymer hydrogels need to be used in combination with other reinforcement materials [32,33].

Kamdem Tamo et al. [34] proposed the 3D printing of bioinspired cellulose nanofibers-filled chitosan hydrogel scaffolds, in which chitosan (CHI) served as a cell growth promoting matrix with balanced hydrophilic properties, while the cellulose nanofibers (CNF) provided the printability and mechanical reinforcement to the final 3D hydrogel construct [34,35]. Cellulose is the main biopolymer found in biomass [36,37,38]. Its crystalline nature gives nanocellulose fibers a compact and oriented microstructure with good mechanical properties [39,40,41,42,43,44]. The addition of CNF able to form a nanofibril network [45] into CHI hydrogels ensured good printability and constructs’ resolution. Three-dimensional printed CHI/CNF hydrogels showed good mechanical performance (with Young’s modulus up to 3.0 MPa, stress at break of 1.5 MPa and strain at break of 75%), anisotropic microstructure and suitable biological responses [34], even at relatively low chitosan concentrations (<5%). To assess the suitability of these material formulations for tissue engineering, fibroblast cells were cultured in such CHI/CNF 3D printed macro-scaffolds. We achieved a good growth and proliferation of fibroblasts within the constructs, showing that the processing and CNF addition did not compromise the chitosan biocompatibility and bioactivity. Thus, the unique mechanical properties of native cellulose fibers offered new strategies for the design of environmentally friendly, high mechanical performance and biocompatible composites for tissue engineering applications [34,35]. Moreover, CNFs are relatively low-cost, abundant and versatile biomaterials that can be used in a wide variety of tissue engineering applications [46,47,48,49], such as skin wound healing and regeneration, cartilage regeneration and drug delivery carriers [50,51]. Despite its excellent mechanical properties, non-toxicity and biocompatibility in vivo, the lack of enzymes capable of hydrolyzing nanocellulose in the human body and its high crystallinity might limit its use for the preparation of resorbable implants [52,53].

Thus, in addition to CNF, to exploit their excellent mechanical properties and biocompatibility [54,55], it is worth evaluating and tuning their biodegradation in vitro and in vivo. Indeed, the mechanical reinforcement of cellulose-based biomaterials should be adapted, accounting for the duration of their resorption in the human body [52,56]. The nanofibers could provide the biomaterial mechanical properties needed in the short term at the macroscopic and cellular levels during tissue regeneration, and the implant should be fully biodegraded and replaced by new tissue in the long term [57,58]. To achieve controlled biodegradation of CNFs, Kamdem Tamo et al. [59] developed cellulase-loaded alginate particles, which serve as carriers for enzyme release, and therefore, sustained biodegradation of cellulose both in homogeneous (cellulose in solution) and heterogeneous conditions (i.e., as suspended cellulose nanofibers). Alginate microparticles with high surface area-to-volume ratio effectively allowed the controlled release of encapsulated cellulase, and thereby, hydrolysis of the CNFs. The relative activity of cellulase encapsulated leveled off at around 60% after one day and practically remained at that value for three weeks [59].

The aim of this work is to develop smart 3D bioprinted hydrogel materials reinforced by cellulose nanofibers (CNFs), useful in tissue engineering, but with controlled biodegradation. The enzymatic biodegradation of the CNFs could be achieved by printing scaffolds by extrusion-based bioprinting of chitosan/CNF inks that incorporate the cellulase enzyme immobilized in nanoparticles. The kinetics of biodegradation of CNFs in enzyme-laden CHI/CNF printed hydrogel composites will be evaluated in vitro, in cell culture studies. To this end, the activity and storage stability of the enzyme in the hydrogel composites, as well as cell viability, will be investigated along the incubation of the enzyme-laden hydrogels in the presence of fibroblast cells.

This work opens up new perspectives in understanding the process of resorption of mechanical functional fiber-reinforced hydrogel composites, for their potential application in vivo as biomaterial implants that aim to support tissue regeneration and are supposed to be biodegraded and fully replaced by the new formed tissue. Cellulose nanofibers’ biodegradation being difficult in vivo often poses some issues in exploiting their excellent mechanical properties and biocompatibility in composites for in vivo applications. To degrade the cellulose nanofibers in the hydrogel scaffolds, the incorporation of the enzyme (i.e., cellulase), which is preliminarily immobilized in an appropriate polymer nanocarrier, contributes both to limiting enzyme denaturation and allowing its controlled release for the sustained degradation of the scaffolds, 3D cell infiltration and controlled formation of new tissue. In addition, the immobilization should protect the enzyme from the gelation chemistry conditions used to achieve stable hydrogel constructs by 3D printing.

## 2. Materials and Methods

### 2.1. Materials

Sodium caseinate (NaCas) was kindly provided by Frédéric Prochazka (Lactips, Saint-Jean-Bonnefonds, France) and was produced from bovine milk.

Chitosan from squid pen chitin was supplied by Mahtani Chitosan (Type: CHITOSAN 144, Batch No. 20120926, Veraval, Gujarat, India). The chitosan molecular weight was determined by size exclusion chromatography (SEC) coupled to multi-angle laser light scattering (MALLS) [60,61,62]. To this end, chitosan solutions at 0.1% (*w*/*v*) were prepared in an acetic acid/ammonium acetate buffer pH = 4.5 (AcOH (0.2 M)/AcONH4 (0.15 M)), which was used as eluent. Then, they were filtered through 0.45 μm pore size membranes (Millipore, Merck KGaA, Darmstadt, Germany). The chromatographic equipment was composed of an IsoChrom LC pump (Spectra-Physics, Santa Clara, CA, USA) connected to a protein pack 200 SW (Waters GmbH, Eschborn, Germany) column and a TSK gel G6000PWXL. A multiangle laser light scattering detector DAWNDSP (Wyatt Technology Europe GmbH, Dernbach, Germany), operating at 632.8 nm, was coupled online to a WATERS 410 differential refractometer. The chitosan number and weight-average molecular weights (Mn and Mw) determined by SEC/MALLS were 4.10 × 10^5^ g/mol (±6.4%) and 6.11 × 10^5^ g/mol (±9.6%) respectively, which yielded a polydispersity index Ip = Mw/Mn = 1.49 (±11.6%).

Cellulose nanofibers (CNFs) of the nanofibrillated cellulose type were obtained from bleached pine sulfite dissolving pulp at the Centre Technique du Papier (CTP, Grenoble, France), by a mechano-enzymatic method adapted from Pääkkö et al. [63]. Before 1 h incubation at 50 °C with a solution of endoglucanase FiberCare R^®^ (Novozyme, Bagsvaerd, Denmark) at pH 5.0, the pulp was refined at 4.5% consistency with a 12” single disk refiner for 25 min. The digested samples were further refined to obtain a cellulose nanofiber suspension with a Schopper–Riegler (SR) number higher than 80 SR and mean fiber length lower than 300 µm. Fiber suspensions at 2% (*w*/*w*) were collected with an Ariete homogenizer (Montigny le Bretonneux, France), involving 1 pass at 1000 bars, followed by 3 passes at 1500 bars. The obtained CNFs displayed a surface charge density of 40–80 mmol/kg and were weakly charged with carboxylate moieties. The mechanical-biological treatments applied to the pulp allowed extracting the cellulosic fibers from the raw material. This is one of the common methods used to obtain a homogenized sample of nanofibrillated cellulose consisting of a network of hairy cellulose nanofibrils of a very high aspect ratio. In our previous works, analysis such as X-ray diffraction, among others, allowed confirming the purity, as well as the high crystallinity, of the cellulose nanofibers obtained as above [34,54,59].

Enzyme cellulase Celluclast 1.5 L from the fungus *Trichoderma reesei* was purchased from Novozyme (Bagsvaerd, Denmark). Celluclast 1.5 L had a protein content of 124 mg/mL and activity of 700 EGU/g. In previous studies, Kamdem Tamo et al. [59] investigated the optimum conditions for maximum activity of this enzyme in studies performed at different pHs, temperatures, with immobilized or free enzyme systems, for cellulose degradation in homogeneous (in solution: solubilized cellulose derivative) or heterogeneous (in suspension: suspended cellulose nanofibers) conditions. For practical applications, an appropriate enzyme activity could be achieved at the temperature and pH conditions of 37–60 °C and pHs 4.5–6.5, respectively [59].

### 2.2. Preparation and Characterization of Cellulase-Encapsulated Chitosan/Caseinate Nanoparticles

The preparation of chitosan/caseinate nanoparticles was performed following a procedure described by Lall et al. [60] in our previous work. Chitosan (CHI) solutions at 0.1% *w*/*w* were prepared by dissolving chitosan powder in an aqueous solution containing a small amount of acetic acid. The latter allowed the protonation of the amine functions of CHI and, consequently, its dissolution in aqueous medium. Similarly, a 0.1% (*w*/*w*) solution of sodium caseinate (NaCas) was prepared by dissolving NaCas powder in distilled water under magnetic stirring. The CHI and NaCas were dissolved by leaving each of the systems under stirring for 24 h. Subsequently, the obtained CHI solution and the NaCas micellar system were filtered through a 0.45 µm pore size membrane (Whatman GmbH, Dassel, Germany) before use.

To prepare cellulase-encapsulated chitosan/caseinate nanoparticles, a proportion of 1 mg of enzyme cellulase to 2.5 mg of chitosan was used for all preparations. Firstly, the enzyme was mixed with the NaCas solution by adding Celluclast 1.5 L into 5 mL of 0.1% *w*/*w* NaCas solution, and the mixture was thoroughly stirred. Afterward, 2.5 mL of the 0.1% *w*/*w* CHI solution was added dropwise to the cellulase/NaCas aqueous mixture. The cellulase/NaCas/CHI mixture was vigorously stirred for 3 min (at about 1000 rpm). In this way, a polyelectrolyte complex (PEC) was formed as a result of the electrostatic interaction between the negatively charged caseinate and the positively charged chitosan. Then, the stirring speed was reduced to a moderate speed (500 rpm) to allow the polyelectrolyte complex (PEC) nanoparticles to harden, and this moderate stirring was maintained for about 30 min. Afterward, the PEC nanoparticles were centrifuged at 14,500 rpm for 25 min. Subsequently, the supernatants were removed, and the NaCas/CHI PEC nanoparticles containing cellulase were washed twice with distilled water to remove the excess free polymer, i.e., the polymer not participating in PEC formation. The sedimented cellulase-encapsulated NaCas/CHI particles were collected for morphological characterizations, study of the kinetics of cellulase release and their use in the processing of biodegradable cellulose nanofiber-filled chitosan hydrogel scaffolds by 3D bioprinting.

#### 2.2.1. Zeta Potential and Hydrodynamic Size of the Nanoparticles

The hydrodynamic size, size polydispersity index and zeta potential of the chitosan–caseinate-based nanoparticles, obtained by polyelectrolyte complex (PEC) formation, were investigated using a Malvern Zetasizer Nano ZS (Malvern Panalytical GmbH, Kassel, Germany), equipped with a 4 MW laser operating at a wavelength of 633 nm. For each measurement, the capillary cell was filled with approximately 1 mL of the chitosan–caseinate PEC colloidal system. A total of three readings were taken for each sample replicate.

#### 2.2.2. Transmission Electron Microscopy (TEM)

The size, shape and surface structure of the nanoparticles were also characterized in their dry state by using transmission electron microscopy (TEM). The centrifugate of the chitosan–caseinate PEC system was washed several times with distilled water. Thereafter, a droplet containing the washed chitosan–caseinate centrifugate (5 µL) was deposited on carbon-coated copper grids (CF400-CU Carbon Film, 400 Mesh Copper grids), which were previously cleaned with plasma to remove surface contamination and make the grids more adhesive toward the sample, and allowed to dry for 1 min. The excess solution was wiped off, and the sample was negatively stained with 3 µL of uranyl acetate for 2 min. The samples were observed with a model Zeiss LEO 912 Omega (Carl Zeiss Microscopy GmbH, Jena, Germany) TEM microscope.

#### 2.2.3. Enzyme Encapsulation Efficiency and Release Kinetics

A cellulase calibration curve was firstly constructed, using cellulase standard solutions of different concentrations, whose absorbance values were measured at the maximum cellulase absorption wavelength of 275 nm with a UV/Visible spectrophotometer (PerkinElmer, Baesweiler, Germany). The cellulase encapsulation efficiency was calculated from the spectrophotometric assay of the supernatant collected after the encapsulation of cellulase in the chitosan nanoparticles. The cellulase encapsulation efficiency was about 88%. The percentage of cellulase released was also determined for different incubation times (1 h, 2 h, 4 h, 6 h, 1 d, 3 d, 6 d, 10 d, 14 d, 17 d, 21 d and 25 d). Initially, the chitosan nanoparticles containing cellulase were introduced into 2 mL of PBS solution, and the system was incubated. After reaching the pre-established incubation time, the supernatant was collected, centrifuged at 14,500 rpm for 10 min, and its absorbance at 275 nm wavelength was measured using the UV/Visible spectrophotometer (PerkinElmer, Baesweiler, Germany). Finally, the percentage of cellulase released was calculated from the cellulase concentration estimated in the supernatant by using the spectrophotometric method. Equations (1) and (2) were used to calculate cellulase encapsulation efficiency and the percentage of cellulase released, respectively.

(1) $$\mathrm{Cellulase}\;\mathrm{Encapsulation}\;\mathrm{Efficiency}\;\left(\%\right)=\frac{\mathrm{Total}\;\mathrm{Amount}\;\mathrm{of}\;\mathrm{Cellulase}-\mathrm{Amount}\;\mathrm{of}\;\mathrm{Free}\;\mathrm{Cellulase}}{\mathrm{Total}\;\mathrm{Amount}\;\mathrm{of}\;\mathrm{Cellulase}}\times 100$$

(2) $$\mathrm{Percentage}\;\mathrm{of}\;\mathrm{Cellulase}\;\mathrm{Released}\;\left(\%\right)=\frac{\mathrm{Amount}\;\mathrm{of}\;\mathrm{Free}\;\mathrm{Cellulase}\;\mathrm{in}\;\mathrm{the}\;\mathrm{Supernatant}\;}{\mathrm{Total}\;\mathrm{Amount}\;\mathrm{of}\;\mathrm{Cellulase}\;\times \;\mathrm{Cellulase}\;\mathrm{Encapsulation}\;\mathrm{Efficiency}\;}\times 100$$

### 2.3. 3D Printing of Cellulose Nanofiber-Filled Chitosan (CHI/CNF) Hydrogel Scaffolds with Entrapped Enzyme

The 3D printing of scaffolds of CHI/CNF containing or not the cellulase enzyme was performed with a 3D-Discovery Evolution bioprinter device (RegenHu Ltd., Villaz-St-Pierre, Switzerland), consisting of an *x*–*y*–*z*-axis positioning system with a tool charger equipped with several printhead stations and a building platform. The 3D structures were printed using a CAD software controlled xyz motion system that guides the tip position. Multilayer square-shaped porous hydrogel structures were 3D printed by microextrusion using a pressure-controlled direct-ink-writing system, with a printing speed of 40 mm s^−1^, applying an extrusion pressure of 30–45 kPa.

Firstly, to obtain the CHI/CNF/cellulase inks, viscous suspensions consisting of solubilized chitosan with dispersed CNFs were prepared. The CHI powder was mixed with the suspension of nanofibrillated cellulose (CNF) at the given composition CHI2%/CNF0.2%. The mixture CHI/CNF suspension (50 mL) was sonicated using a SONOPULS ultrasonic homogenizer (Bandelin electronic GmbH, Berlin, Germany) for 5 min at 40% amplitude. Subsequently, the mixture was transferred to a mechanical stirrer, and a stoichiometric amount of acetic acid was added to completely solubilize the chitosan contained in the mixture. The mixture was left under mechanical stirring for 24 h in a closed reactor. Afterward, the viscous CHI/CNF suspension was collected for further use. Cellulose nanofiber-filled CHI viscous suspensions containing 2% *w*/*w* of CHI and 0.2% *w*/*w* of CNF (CHI2/CNF0.2) were then prepared.

Before processing the enzyme-entrapped CHI/CNF hydrogel scaffolds by 3D printing, the cellulase-entrapped CHI/NaCas nanoparticles (in their wet state), obtained as above, were carefully mixed for 1 min with the CHI/CNF viscous ink, by means of a spoon spatula. For the preparation of cellulase-loaded inks, the collected chitosan–caseinate hydrogel nanoparticles containing ~2 mg of encapsulated cellulase, or a similar amount of free enzyme, were mixed with 3 mL of the CHI2/CNF0.2 viscous suspension. After fixing the cartridge containing the CHI/CNF/Cellulase ink to the 3D printer printhead, 3D scaffolds were directly printed in cell culture wells containing a coagulation bath of 5% *w*/*v* sodium tripolyphosphate TPP dissolved in phosphate-buffered saline PBS (pH 7.4). The composite inks were printed through precision conic dispense tips with an inner diameter of 580 µm (Nordson EDS, Feldkirchen, Germany). After 2 min gelation, the TPP/PBS solution was removed from the wells, and the printed CHI/CNF/enzyme scaffolds were washed using a PBS solution for 1 min. To investigate the degradation of CNFs, two-layered square scaffolds of 2 × 2 cm^2^ dimension were printed, with 0.85 mm distance between the printed hydrogel filaments. The 3D printing of CHI/CNF scaffolds containing the non-encapsulated enzyme (i.e., printed with the ‘free’ enzyme), as well as of reference scaffolds of CHI/CNF without the enzyme, was carried out following the same procedure as described above. For the 3D printing of scaffolds containing the free enzyme, the amount of free enzyme added to the CHI/CNF viscous ink was equivalent to that used when processing enzyme-encapsulated nanoparticles.

### 2.4. Enzymatic Biodegradation of Cellulose Nanofibers Contained in 3D Printed CHI/CNF Hydrogels Used in Cell Culture Studies—Enzyme Activity Assay

Prior to incubation with cells of the 3D printed CHI/CNF/enzyme hydrogel scaffolds, the latter were washed with a sterile solution of PBS (pH 7.4) to remove the excess of TPP-based gelation solution. Afterward, fibroblast cells in the culture medium were seeded on the 3D printed CHI/CNF hydrogels, considering an average of 100,000 cells per well. Sterile polystyrene Petri dishes of 35 mm diameter were used (SARSTEDT AG & Co. KG, Nümbrecht, Germany). Then, the DMEM cell culture medium was added to adjust the total volume of the solution to 2 mL. Finally, the scaffolds were incubated with the cells at 37 °C.

#### Cellulase Activity Assay

The activity of the enzyme cellulase contained in the 3D printed CHI/CNF/enzyme scaffolds, either printed with an enzyme previously encapsulated in the CHI/NaCas nanoparticles (entrapped enzyme) or directly as a free enzyme, was evaluated at different incubation times by measuring the quantity of sugar-reducing ends produced after enzymatic hydrolysis of cellulose constituting the CNFs. To this end, at different times, the supernatant of each cell culture well, which also contained the printed scaffold, was collected (2 mL of supernatant) and placed separately in different glass vials of 10 mL volume. Afterward, 3 mL of a 0.044 M dinitrosalicylic acid (DNS) was added per vial. The prepared mixture was heated for 5 min in a boiling water bath, at which time a red-brown color developed due to the reduction in DNS by the produced sugar-reducing ends into 3-amino-5-nitrosalicylic acid, which could be quantified by UV spectrophotometry at 540 nm wavelength of maximum absorbance. The unit of cellulase enzyme catalytic activity (IU) is defined as the amount of cellulase that hydrolyzes the cellulosic substrate to produce 1 µmol of glucose per minute.

The enzyme activity in the 3D printed CHI/CNF/enzyme hydrogels under cell culture studies was determined at incubation times of 1 h, 2 h, 3 h, 6 h, 1 d, 3 d, 5 d, 8 d, 10 d, 12 d and 14 d. As normalization, at similar times, the activity of both the encapsulated and the free enzyme in the systems, which only contained the cell culture medium and fibroblast cells, was also determined by the DNS method. To subtract any influence of the cell culture medium compounds and the effect of the cells, the number of sugar-reducing ends, and consequently, the enzyme activity values determined when incorporating the 3D printed scaffolds, were subtracted by those values obtained only for the cell culture medium plus the cells.

### 2.5. Fluorescence Imaging of Printed CHI/CNF Hydrogel Filaments Containing FTIC-Labeled Cellulase Encapsulated in Chitosan–Caseinate Nanoparticles

Enzyme staining with fluorescein isothiocyanate (FITC) and fluorescence microscopy were used to evaluate the dispersion and distribution of the cellulase enzyme in the biomaterial after the processing of enzyme-loaded hydrogels by 3D printing. To this end, the cellulase was FITC labeled following a previously reported protocol [59]. FITC-labeled cellulase was collected to prepare nanoparticles with the encapsulated FTIC-labeled enzyme. The latter was encapsulated in chitosan/caseinate polyelectrolyte complex (PEC) nanoparticles, following the above-described procedure to prepare cellulase-encapsulated CHI-NaCas nanoparticles (see Section 2.2). Afterward, hydrogel filaments were printed in a TPP/PBS solution (crosslinking solution) using, as ink, a viscous solution CHI2%/CNF0.2% containing FITC-labeled cellulase encapsulated in the CHI-NaCas nanoparticles. The hydrogel filaments were left to harden for 2 min in the TPP/PBS solution. Afterward, the TPP/PBS solution was removed, and the hydrogel filaments were rinsed twice in a PBS solution. Subsequently, the hydrogel filaments were incubated at different times in PBS at 37 °C. Fluorescence microscopy images of the printed hydrogel filaments, which were supposed to contain the FTIC-labeled cellulase, were taken at the different incubation times: 30 min; 1 d; 3 d; 5 d; and 7 d. A confocal laser scanning microscope was used, which was equipped with detectors and filter sets for fluorescence monitoring.

### 2.6. Scanning Electron Microscopy (SEM) of Freeze-Dried 3D Printed CHI/CNF Scaffolds

To characterize the morphology of the initial and biodegraded 3D printed CHI/CNF scaffolds, both CHI/CNF without the enzyme and CHI/CNF/cellulase hydrogels containing the encapsulated cellulase were freeze dried either immediately after the 3D printing process (initial) or after a given time of incubation (biodegraded), for example, after 21 days of enzymatic hydrolysis. Then, to observe the microstructure of the freeze-dried scaffold filament cross-sections, they were gold sputtered in a Polaron SC 7640 (VG Microtech, East Sussex, UK) and observed with a scanning electron microscope FIB SEM (FEI Scios 2 Dual Beam, Hillsboro, OR, USA) at a voltage of acceleration of 20 or 5 kV.

### 2.7. Cell Culture of Fibroblasts in the 3D Printed Hydrogel Scaffolds

To assess the suitability of 3D printed enzyme-laden CHI/CNF hydrogels for cell proliferation, fibroblast cells were cultured in the 3D hydrogel scaffolds. For comparison purposes, cell culture was also performed on the 3D printed scaffolds that did not contain any enzyme. Cultures of the 3T3 cells developed using NIH Swiss mouse embryo fibroblasts were performed (murine fibroblast, strain: NIH/Swiss). Cells were grown in T75 cell culture flasks (75 cm^2^) (Sarstedt, Nümbrecht, Germany). Cells were grown in Dulbecco’s Modified Eagle’s Medium (DMEM) supplemented with 2 mM L-glutamine and 10% fetal bovine serum (FBS) (Gibco, Thermo Fisher Scientific, Leicestershire, UK) at 37 °C in a humidified atmosphere at 5% CO_2_ for 1 week. At 90% confluence, cells were rinsed twice with phosphate-buffered saline (PBS) (Gibco, Thermo Fisher Scientific, Leicestershire, UK) followed by stripping with trypsin/ethylenediamine tetraacetic acid (EDTA) for 5 min and neutralization with the corresponding cell culture medium. After detachment, cells were centrifuged in a centrifuge for 5 min at 110 g (Rotor F-45-30-11, Eppendorf 5417R, Hamburg, Germany). The supernatant was discarded, and the cells were diluted in the culture medium. The 3D printed CHI/CNF hydrogel scaffolds were placed in cell culture plates for further use for cell growth. For this purpose, a suspension of the cells in the culture medium, containing 500,000 cells, was added onto the 3D printed CHI/CNF hydrogel scaffolds. NIH/3T3 fibroblasts seeded in triplicate in scaffolds were maintained at 37 °C in 5% CO_2_ incubation. Cells seeded in empty wells (i.e., without 3D printed hydrogels) were used as a control.

#### Live/Dead Cell Viability Assay

Cell viability was assessed to obtain information on the survival and growth of fibroblasts in CHI/CNF hydrogel scaffolds containing cellulase encapsulated in chitosan nanoparticles. Cell viability was investigated by fluorescent staining with a live/dead staining kit: calcein AM/ethidium-homodimer-1, LIVE/DEAD™ viability/cytotoxicity kit, (Thermo Fisher Scientific, Leicestershire, UK). After 1, 2 and 3 days of cell culture in the 3D hydrogel scaffolds, the 3D scaffolds were washed with Hank’s balanced salt solution (HBSS, Gibco, Thermo Fisher Scientific, Leicestershire, UK), and then, the LIVE / DEAD solution was added to each sample. The washed scaffolds were incubated for 15 min. After staining, the wells were imaged using a laser scanning confocal microscope. As a LIVE indicator, calcein AM labels cell cytoplasm with green fluorescence; and as a DEAD indicator, the ethidium-1 homodimer labels the cell nucleus with red fluorescence.

## 3. Results

### 3.1. Cellulase-Encapsulated Chitosan–Caseinate PEC Nanoparticles and Kinetics of Controlled Release of Cellulase

**Figure 1:**
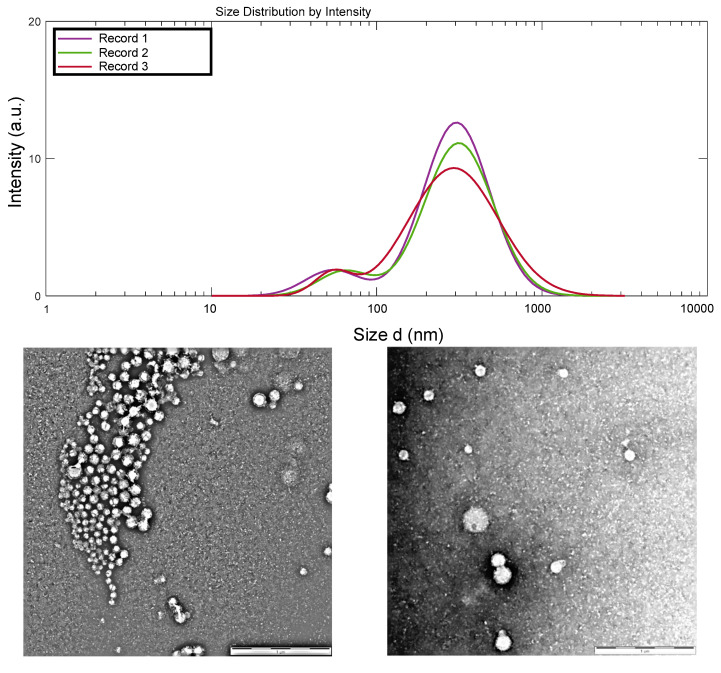

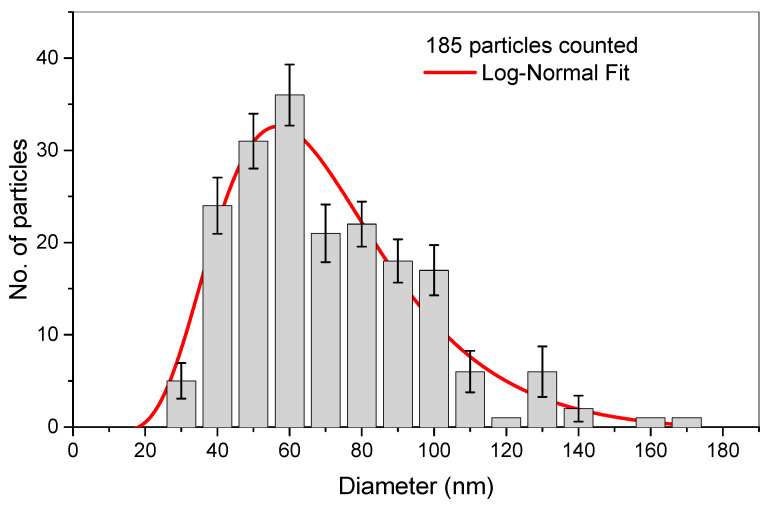
(**Top**) Hydrodynamic size distribution of the chitosan–caseinate nanoparticles, obtained by dynamic light scattering analysis. (**Middle**) TEM micrographs of chitosan–caseinate nanoparticles (scale bars: 1 μm). (**Bottom**) The corresponding number–average size distribution histogram of the obtained dried nanoparticles and adjusted log–normal distribution.

From the dynamic light scattering (DLS) measurements, an average hydrodynamic size and a polydispersity index of 220.8 nm and 0.326 were determined, respectively (Figure 1), after the addition of 2.5 mL of the here used 0.1% (*w*/*w*) of high molecular weight CHI of low degree of acetylation onto 5 mL of 0.1% (*w*/*w*) caseinate solution, to achieve polyelectrolyte complex nanoparticles. The obtained hydrodynamic size distribution curve is displayed in Figure 1. Specifically, a bimodal size distribution was observed, but with 93% of the nanoparticles (Peak 1) presenting a mean hydrodynamic size around 329.1 nm and only 7% (Peak 2) revealing a hydrodynamic size around 55.9 nm. The nanoparticles remained stable with no formation of aggregates due to a determined zeta potential value of +46 mV, far enough from the zero value as to allow stabilization of the polyelectrolyte colloidal system.

The transmission electron microscopy (TEM) images of Figure 1 show the morphology of the CHI–caseinate nanoparticles collected after centrifugation, which were, in addition, dried during the sample preparation for TEM observations. The nanoparticles showed a spherical shape with a porous morphology, which should be related to the open micellar character of the polyanion caseinate. In the nanoparticles, these micelles (associated or not with the cellulase enzyme) are supposed to be in the inner region of the nanoparticles (as previously described by Lall et al. [60]), then decorated at their surface by the (highly deacetylated) polycation chitosan, which was added onto the caseinate micellar dispersion during nanoparticle processing. The TEM micrographs also show that smaller particles were achieved with an average diameter of about 67 nm (dry state) and a relatively narrow size distribution (Figure 1).

#### Cellulase Enzyme Entrapment Efficiency and Its Release from CHI-NaCas Nanoparticles

The cellulase entrapment efficiency was calculated from the UV spectrophotometric analysis at 275 nm wavelength of the supernatant collected after the encapsulation of cellulase in the chitosan–caseinate (CHI-NaCas) nanoparticles. The cellulase encapsulation efficiency, calculated using the formula presented in Equation (1), was about 88%.

**Figure 2:**
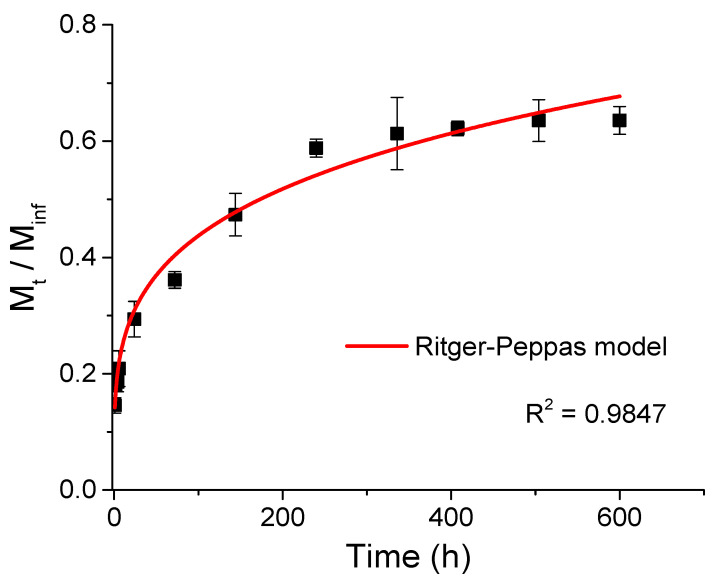
Cellulase release profile from chitosan–caseinate nanoparticles in phosphate-buffered saline solution PBS (pH 7.4) at a temperature of 3 7 °C. Ritger–Peppas model fitting curve in the time ranges up to 600 h (25 days).

Figure 2 shows the kinetics of cellulase release from chitosan–caseinate nanoparticles, performed in a buffer medium (PBS pH 7.4). A gradual release of cellulase from CHI-NaCas nanoparticles was observed during the first 240 h (10 days). Afterward, the release of cellulase reached a plateau. Cellulase release during the first hour was about 15% and reached about 60% after 240 h. This could indicate that if these enzyme-laden nanoparticles were introduced into a cellulose-containing hydrogel with the aim of degrading cellulose by the released cellulase, the degradation could first be controlled by the enzyme delivery, i.e., progressive, during the first hours of incubation, before stabilizing after about ten days.

The kinetics of cellulase release in the early stages may be determined by the pre-establishment of molecular interactions between the caseinate phosphoprotein and cellulase before the polyelectrolyte association with chitosan. Caseinate comprises four peptides, which are similar in their amphiphilic character. The hydrophilic and hydrophobic regions of caseinate show block distribution in the protein chain. Casein peptides bear a negative charge on their surface due to phosphorylation. Thanks to these amphiphilic properties, under the appropriate conditions, caseinate molecules agglomerate into spherical micelles [64]. This suggests that the physicochemical properties of the release medium (pH and ionic strength) will have a major impact on these kinetics [60].

The cellulase release data from chitosan–caseinate nanoparticles in PBS could be modeled with the Ritger–Peppas empirical release kinetics model, as shown in Figure 2. The Ritger–Peppas model equation used reads as follows:

(3) $$\frac{M_{t}}{M_{\infty }}=k\cdot t^{n}$$

where *M*_∞_ is the amount of released cellulase at the equilibrium state, *M*_t_ is the amount of cellulase released at time *t*, *k* is the release velocity constant (incorporating structural modifications and geometrical characteristics of the system), *n* is the exponent related to the enzyme release mechanism. The obtained R^2^ value was close to 1 (R^2^ = 0.9847 and Adj. R^2^ = 0.9832), and a low SSR value (close to 2.40) was obtained for the adjustment with the Ritger–Peppas model Equation (3). The fit yielded *k* = 0.139 ± 0.009 h^−1^ and *n* = 0.25 ± 0.01 as the diffusional exponent.

Based on the apparent value of *n*, it is usual to establish a classification of behavior observed. Assuming the spherical geometry of the CHI-NaCas nanoparticles, several *n* ranges correlate as follows for the diffusion types: (a) *n* < 0.43 for pseudo-Fickian, (b) *n* = 0.43 for Fickian (case I) diffusion, (c) 0.43 < *n* < 1.00 for anomalous (non-Fickian) transport and (d) *n* = 1.00 for zero-order release (case II) [61,62,65,66]. Rietger and Pepas [67] also pointed out the importance of the size distribution of particles in the apparent release and values of *n* from the phenomenological equation (3). Such distribution effects result in apparent values lower than 0.43, which is valid for a monodispersed collection of spheres, and an evaluation of Equation (3) on the first 60% release.

In the Fickian model (Case I), *n* = 0.43, the release of the active substance is controlled by diffusion. Polymer relaxation is much faster than water diffusion, and the diffusion is followed by an instantaneous response of the system, resulting in Fick’s behavior. The absorption equilibrium in exposed surface of the polymer system may occur rapidly, also leading to time-dependent binding conditions. The kinetics of this phenomenon is also characterized by diffusivity [68,69,70]. 

In the initial work by Ritger and Pepas [67], the release of solute by diffusion from non-swellable spheres is numerically studied, and the $\frac{M_{t}}{M_{\infty }}\;$ ratio is modeled according to the radius of spheres *r* and diffusion coefficient *D* of the solute throughout the sphere. In particular, $\frac{M_{t}}{M_{\infty }}\;$ reaches 0.5 (‘mid-release’) when the dimensionless time $\tau =\frac{Dt}{r^{2}}$ is close to 0.04. In Figure 2, the mid-release is obtained at about 20 h (*t*_1/2_ = 7.2 × 10^4^ s). If a radius of approximately 50 nm is chosen as the radius of the sphere (see Figure 1), then the diffusion coefficient *D* should be in the order of $\frac{\tau r^{2}}{t_{1/2}}$~1.4 × 10^−21^ m^2^/s~1.4 × 10^−17^ cm^2^/s, which is an extremely low value in comparison to the diffusion of macromolecules of various molar masses, for example, in polyacrylamide (PAM) hydrogels, where *D* values ranging from 5 × 10^−6^ to 10^−8^ cm^2^/s are obtained and modeled using various techniques and physical approaches [71].

As a result, the cellulase in the casein/chitosan particles can be considered as tightly entrapped, and its release should not be considered as conditioned by diffusion but as strongly slowed down thanks to the hydrophobic and/or electrostatic interactions within the casein micelles and the casein/chitosan complexes. This results in long release times, which can be leveraged for the protection and sustained release of the enzyme.

### 3.2. 3D Printed CHI/CNF Scaffolds Containing Cellulase Encapsulated in Chitosan Nanoparticles

**Figure 3:**
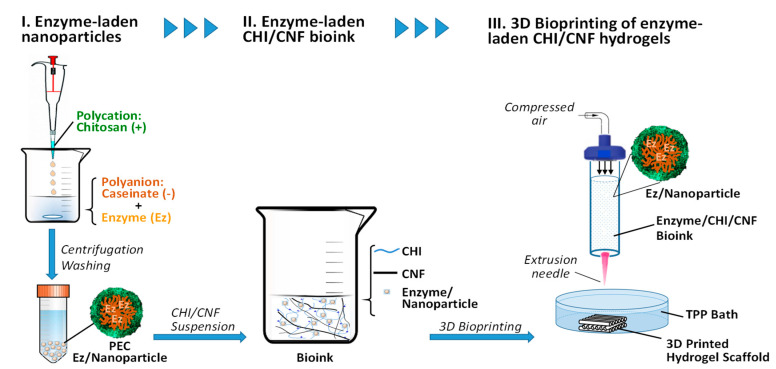
Schematic representation of the processing of enzyme-laden CHI/CNF hydrogel scaffolds by 3D printing of CHI/CNF bioinks containing enzyme (Ez) entrapped polyelectrolyte complex (PEC) nanoparticles.

The scheme in Figure 3 illustrates the preparation of CHI/CHF/enzyme scaffolds by 3D printing. Cellulase was first encapsulated in chitosan–caseinate (CHI-NaCas) nanoparticles by adding the CHI solution dropwise to an excess of a caseinate solution containing the cellulase enzyme. After 30 min of nanoparticles hardening, the system was centrifuged, and the nanoparticles containing the cellulase were collected and mixed with the CHI/CNF composite ink (CHI2%/CF0.2%), as described in detail in Section 2. Materials and Methods. Finally, the cellulase-laden 3D scaffolds were printed.

#### 3.2.1. Microstructure Characterization of 3D Printed Cellulase-Entrapped CHI/CNF Hydrogels

**Figure 4:**
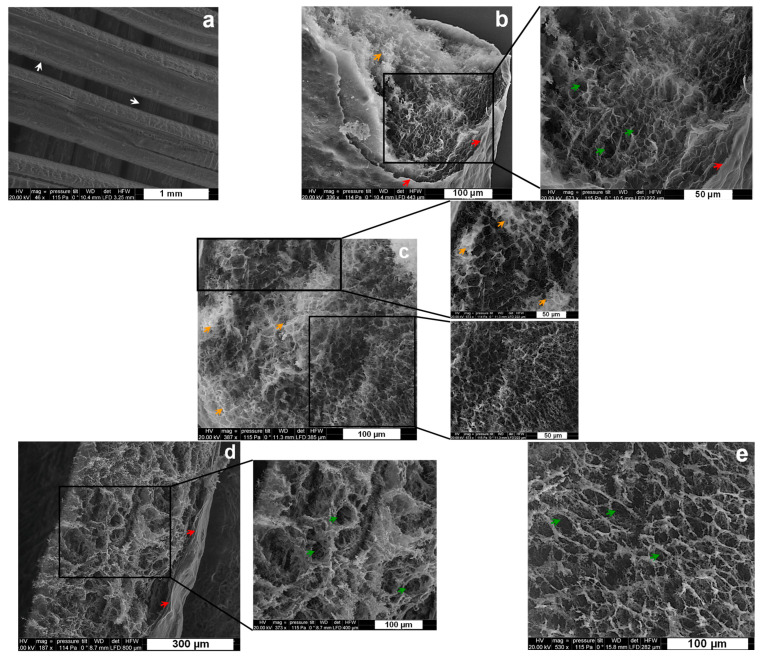
SEM micrographs of 3D printed hydrogel scaffolds after freeze drying. (**a**–**c**) CHI2/CNF0.2 with entrapped cellulase: (**a**) overview of the surface of the filaments in the different sublayers of the scaffold; (**b**,**c**) filament cross-section surface at different magnifications, showing an inner spongy-like network of the filament covered by a skin-like shell (Red arrows) at the outer surface. (**d**,**e**) CHI2/CNF0.2 reference scaffold without cellulase: filament cross-section surface at different magnifications, also showing an inner spongy-like network of the filament covered by a skin-like shell (Red arrows) at the outer surface. Arrows: White: borderline of filaments printed in different scaffold sublayers; Red: skin-like shell structure of the outer surface of the filaments; Green: pores distributed in the whole inner microstructure of the filaments; Orange: brightness spots probably related to some remaining salt, such as the sodium acetate, formed after mixing chitosan-acetate and sodium-caseinate to prepare the chitosan–caseinate PEC nanoparticles.

CHI/CNF 3D printed scaffolds, with incorporated cellulase-entrapped chitosan–caseinate nanoparticles, were immediately freeze dried and analyzed by SEM. Figure 4a shows the distinct extruded filaments of uniform thickness of approximately 400 µm. At a higher magnification, Figure 4a–c shows the internal microstructure of the filaments constituting the scaffolds printed from bioinks in which the immobilized cellulase was incorporated.

These images highlight the interest in a freeze-drying step generating a porous structure with interconnected pores, possibly allowing a good diffusion of the enzyme molecules, promoting the biodegradation of CNFs in a rehydrated matrix, as well as the migration and proliferation of cells. In addition to this, the SEM images show a good integration of tcellulose nanofibers and enzyme-containing chitosan-casein nanoparticles in the chitosan matrix, where both nano-objects are no longer distinguishable in the chitosan matrices in the SEM images. For comparison, Figure 4d,e displays the inner microstructure of freeze-dried filament of 3D printed CHI/CNF scaffold, which was printed without any incorporated enzyme. Comparing Figure 4d,e with the SEM images of the scaffolds containing the cellulase enzyme obtained at the same magnifications (Figure 4b,c), we can note that the presence of the immobilized cellulase in the scaffolds practically does not affect the microstructure of the resulting lyophilizates (and possibly neither the microstructure of their parent hydrogel scaffolds).

#### 3.2.2. Morphology Evolution of the CHI/CNF/Entrapped-Cellulase Scaffolds during Enzyme Release and Biodegradation

**Figure 5:**
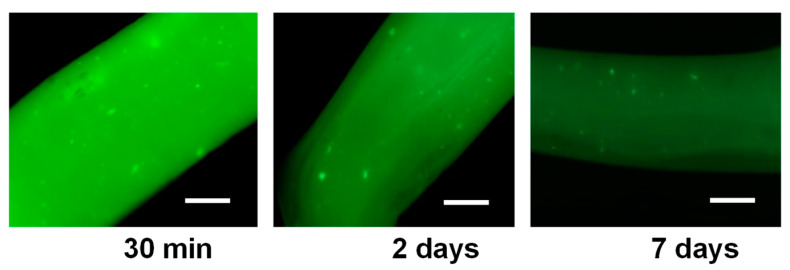
Fluorescence photos of printed filaments of CHI/CNF/Entrapped-cellulase filaments containing the cellulase stained with fluorescein isothiocyanate (FITC), after different times of incubation at 37 °C in PBS (pH 7.4). Scale bars: 200 µm.

Figure 5 shows the fluorescence micrographs of printed hydrogel filaments, originating from a bioink containing FITC-labeled cellulase encapsulated in CHI-NaCas nanoparticles mixed with CHI/CNF viscous suspensions. These pictures were taken after 30 min, 2 or 7 days after incubation of the printed enzyme/CHI/CNF filaments in PBS solution. The initial intense green color reveals that the filaments contain an appreciable and homogeneously distributed amount of enzyme entrapped in the printed hydrogel, probably with modest leakage of the enzyme during the printing process thanks to cellulase/casein/chitosan interactions. After 2 days, the decrease in fluorescence intensity may result from the release of the enzyme by the CHI/NaCas nanoparticles and then diffusion into the PBS media, in which the filaments were suspended. The observation of the gradual intensity decrease is illustrated for day 2 and day 7 in Figure 5. The enzyme release mechanism quantified above for the isolated nanoparticles and then observed for the enzyme-loaded nanoparticles incorporated in printed hydrogels should promote the sustained release and diffusion of the enzyme through the chitosan hydrogel matrix to reach the CNF network to be enzymatically biodegraded.

**Figure 6:**
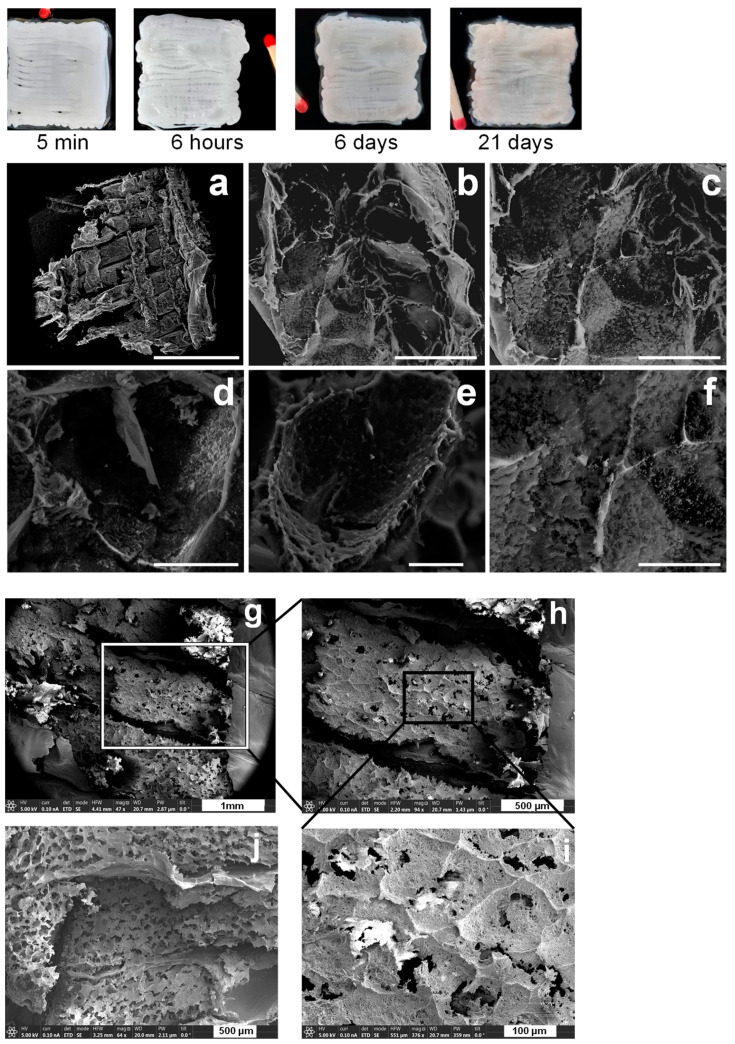
(**Top**) Macroscopic images of 3D printed CHI/CNF/Entrapped-cellulase hydrogel scaffolds evolution after printing and further incubation in PBS (pH 7.4) at 37 °C, allowing sustained CNF enzymatic biodegradation, as shown for different times: 5 min, 6 h, 6 d and 21 d. (**Middle and Bottom**) SEM micrographs of the microstructure of corresponding freeze-dried scaffolds obtained after enzymatic degradation for 21 days (**a**–**f**) (scale bars: (**a**) 3 mm (overview), (**b**) 200 μm, (**c**,**d**) 100 μm, (**e**,**f**) 50 μm) and for 14 days (**g**–**j**), at different magnifications and filament positions.

The morphological evolutions of the printed hydrogel scaffolds before and after enzymatic biodegradation are displayed in Figure 6, showing CHI/CNF hydrogels printed with cellulase immobilized in CHI-NaCas nanoparticles, taken after 5 min, 6 h, 6 d and 21 d of incubation in PBS (pH 7.4) at 37 °C. The SEM micrographs in Figure 6a–f display the morphology of the printed scaffolds and the inner microstructure of the printed filaments (once the constructs were freeze dried) after 21 d of incubation to allow enzymatic degradation of the cellulose nanofibers. The image of 3D scaffold taken 5 min after the printing shows the initial microstructure with a well-defined and uniform extrudate (filament) shape. Here, again, the homogeneity observed on these 3D printed samples implies a uniform dispersion of cellulose nanofibers within the chitosan hydrogel. In the macroscopic photo of the biodegraded freeze-dried scaffold, for example, after 21 d of enzymatic hydrolysis (Figure 6a–f), the scaffold is appreciably degraded with irregular filaments. This degradation is illustrated in the SEM images of the scaffold cross-sections where especially the inner microstructure of the filaments shows large pores, practically holes within the monofilaments, distinguished by the loss of the initial homogeneous spongy-like microstructure of the scaffold (as shown above in Figure 4). Thus, it seems that after degradation of the nanocellulose, the initial microstructure of the composite scaffold resulting from the cellulose nanofiber network entrapped in the chitosan hydrogel matrix easily breaks, a phenomenon that is more emphasized after freezing drying, and the scaffolds do not display the spongy-like morphology anymore, which was indeed present in the initial lyophilizate (Figure 4a). For comparison, the SEM images of the freeze-dried scaffold obtained after 14 d of enzymatic degradation are also illustrated in Figure 6g–j, in which that initial inter-porous membranous network was also partly destroyed, and some broken parts and holes are observed in the inner microstructure, but as expected, the disintegration of the scaffold after 14 d was not as advanced as after 21 d of enzymatic hydrolysis (Figure 6a–f).

### 3.3. Suitability of Biodegradable 3D Printed Cellulase/CHI/CNF Hydrogel Scaffolds for Fibroblast Cell Culture

**Figure 7:**
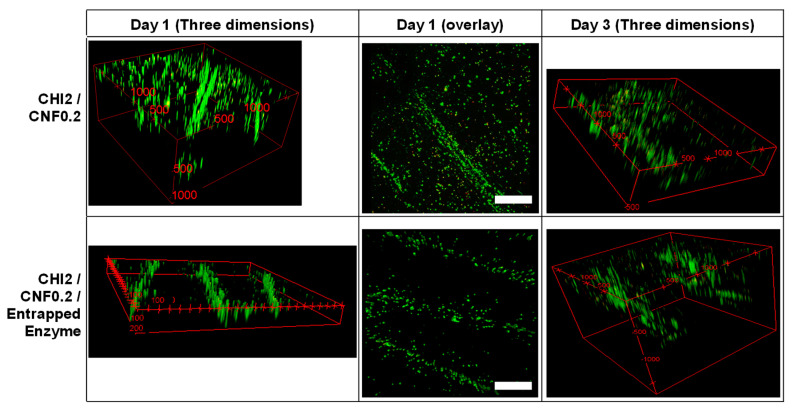
LIVE/DEAD assay confocal laser scanning microscopy (CLSM) images after culture of fibroblast cells in 3D printed CHI/CNF or CHI/CNF/encapsulated cellulase hydrogel scaffolds. LIVE indicator: green fluorescence; DEAD indicator: red fluorescence. Scale bars (Day 1, overlay): 500 μm. Three-dimensional frames scale: CHI2/CNF0.2, Day 1) x × y × z: 2.0 × 2.5 × 1.0 mm^3^, CHI2/CNF0.2, Day 3) x × y × z: 2.0 × 2.5 × 0.5 mm^3^; CHI2/CNF0.2/Entrapped Enzyme, Day 1) x × y × z: 2.3 × 2.3 × 0.2 mm^3^; CHI2/CNF0.2/Entrapped Enzyme, Day 3) x × y × z: 2.0 × 2.0 × 1.5 mm^3^.

**Figure 8:**
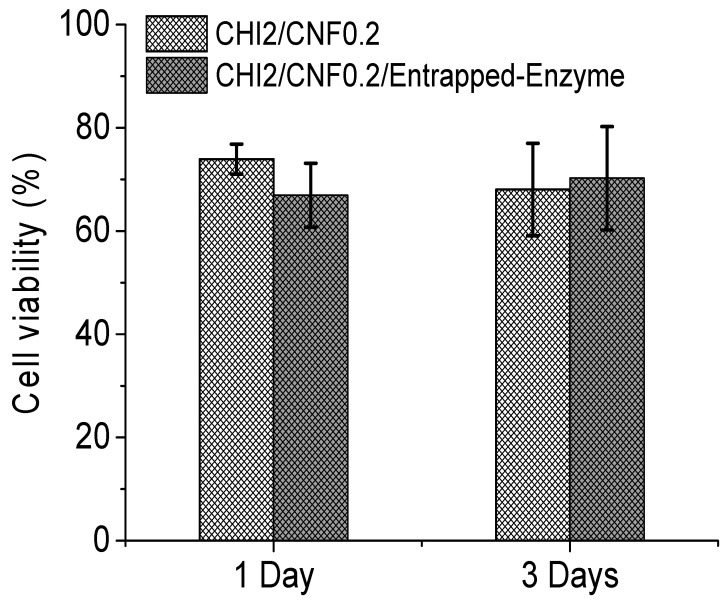
Cell viability at the different days for NIH 3T3 fibroblasts cultured on the 3D printed cellulase-laden cellulose nanofiber-filled chitosan hydrogels and on the printed CHI/CNF reference hydrogels containing no enzyme.

In order to evaluate the biocompatibility and the bioactivity of the 3D printed hydrogels containing the cellulase encapsulated in chitosan/caseinate nanoparticles, in vitro fibroblast cell cultures on 3D printed scaffolds were carried out for 1 and 3 days. To qualitatively assess cell viability, live–dead assays were performed. Figure 7 shows the live/dead assay fluorescence images of the constructs, 1 and 3 days after culturing the cells on the printed scaffolds. For comparison purposes, fibroblast cells were also cultured on printed scaffolds without containing enzyme. After the initial 24 h of culture, a high number of live cells were observed in the 3D printed hydrogel scaffolds with very few dead cells, which showed excellent biocompatibility of 3D printed CHI/CNF hydrogels scaffolds, containing or not encapsulated cellulase enzyme. The results are quantified in Figure 8. Live cells were homogeneously distributed on the filaments, especially more on their surface, and after 3 days of incubation, they infiltrated more the inner part of the filaments, showing 3D cell ingrowth, probably related to the formation of macropores due to the controlled enzymatic degradation of the scaffolds. For all times, fibroblast cells adhered to the 3D printed hydrogels and initially expanded following the direction of the outer surface of the deposited hydrogel filament, with higher cell concentration there at the interstice between the filaments.

The histogram in Figure 8 reports the cell viability (in percentage) recorded in CHI/CNF 3D printed scaffolds containing cellulase entrapped in chitosan–caseinate nanoparticles compared to the cell viability recorded in scaffolds not containing cellulase. Cell cultures on both types of 3D printed scaffolds were performed for 1 and 3 days. The presence of the enzyme does not affect the proliferation of cells in the scaffolds, since the number of living cells recorded after 3 days of culture in the scaffold containing the encapsulated enzyme is greater than the number of cells recorded in the scaffold after 1 day of culture in the scaffold of the same composition. In addition, after 3 days of incubation, the viability of the cells in the scaffolds containing the cellulase is slightly higher (not statistically significant difference) than that obtained 3 days after the culture of the fibroblast cells in the scaffolds not containing the enzyme. As a conclusion, the presence of cellulase in the 3D printed scaffolds promotes the biodegradation of CNFs in the composite hydrogel matrices without compromising the biocompatibility of chitosan and cellulose nanofibers.

#### Kinetics of Enzymatic Biodegradation of Cellulose Nanofibers in 3D Printed CHI/CNF/Entrapped-Cellulase Scaffolds in Cell Culture Studies

**Figure 9:**
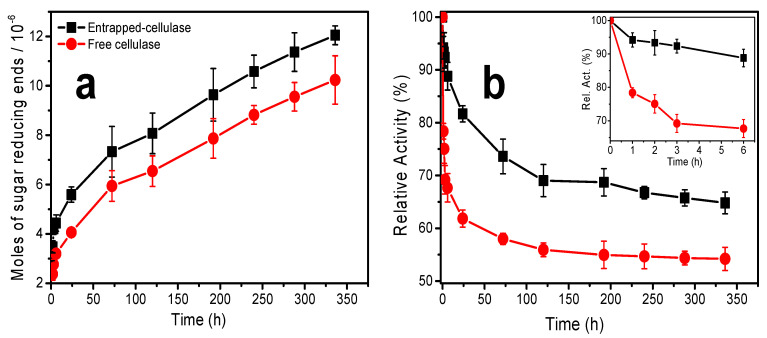
(**a**) Moles of sugar-reducing ends (RE) produced after CNF enzymatic hydrolysis by entrapped and free cellulase at different times; and (**b**) estimated storage stability of entrapped and free cellulase.

Figure 9a shows the amount (mol) of sugar-reducing ends (RE) produced during the hydrolysis of cellulose nanofibers at different incubation times in 3D printed CHI/CNF scaffolds containing the enzyme, additionally incubated with fibroblast cells. The moles of sugar-reducing ends were quantified by the DNS method; their production occurred over the long-studied period of incubation. The number of moles of RE gradually increased with incubation time. This shows that the activity of the enzyme is preserved throughout the degradation process (during the 336 h of enzymatic hydrolysis) and that the enzyme is effectively released within the hydrogel from the casein/chitosan PEC nanoparticles. This is in line with the cellulase release profile of the nanoparticles in the PBS (pH 7.4) solution, where the amount of cellulase released increases gradually during the first 2 weeks of incubation (Figure 2).

Figure 9b shows the activity of encapsulated and free cellulase for different times of degradation of the cellulose nanofibers dispersed in the 3D printed scaffolds. The obtained maximum activities are above 65% and 50% for cellulase preliminarily encapsulated in chitosan–caseinate nanoparticles and free cellulase, respectively. The insert in Figure 9b highlights the activity profile of the free and encapsulated cellulase at short times, from 1 to 6 h of incubation. It can be noted that the activity of the free cellulase drops by approximately 25% after the first hour and 35% after six hours of incubation. From the investigations carried out on the biodegradation of cellulose nanofibers in a solution by free or encapsulated cellulase [59], it was found that cellulase activity in a basic medium was markedly low, with a maximum of activity obtained at pH 4.8 [59]. The widespread practical applications of the free enzyme are limited because of its hydrophilic nature and low stability in various pH and temperature ranges [59,72]. On the other hand, the relative activity of the cellulase encapsulated in the nanoparticles varied very little during the first hours of incubation. It was about 88% after 6 h of incubation. Thus, in the short term, the encapsulation of the cellulase in the chitosan–caseinate nanoparticles helped protect the cellulase from interactions with the substrate components and environmental changes, thus maintaining the stability and functionality of the cellulase.

During the long-term enzymatic hydrolysis, the enzymatic activity of the cellulase was also significantly preserved by encapsulating the enzyme in the chitosan–caseinate nanoparticles. The enzyme activity of the entrapped cellulase achieved a plateau at 120 h and practically remained constant up to 192 h (8 days)—then, with further slow decay and achievement of 65% activity at 336 h (14 days). The relative activity of the free enzyme initially fell very fast and, also at 120 h (5 days), a plateau was achieved, and the activity practically remained unvaried up to 336 h, at which time, an activity of 55 % was determined. These results are comparable to those obtained during the study of the biodegradation of cellulose nanofibers in suspension by cellulase encapsulated in dispersed alginate microparticles [59]. In the present work, the non-immobilized enzyme (free enzyme) should, on the one hand, be less stabilized and, on the other hand, diffuse faster out of the hydrogel and become ineffective, whereas the entrapped enzyme should contribute to the stabilization of the enzyme, which, in addition, might be released slowly, which contributes to a relatively higher amount of the enzyme available for cellulose biodegradation at longer times. These results highlight the fact that in tissue engineering, which integrates the use of cellulose nanofibers as a nanomaterial to reinforce the mechanical properties of the 3D hydrogel scaffold useful for cell growth and proliferation, the biodegradation of the biomaterial can be tuned in vitro during the manufacturing of the implant, where the first support is needed, until the new tissue forms, and eventually, the implanted biomaterial is resorbed. Such strategy would also open up new perspectives for in vitro preparation of neotissues with minimal burden of biomaterials, by means of in vitro controlled degradation.

In our study, the enzymatic degradation could be explained by a mechanism based on enzymatic sustained release, allowing the gradual degradation of cellulose nanofibrils dispersed within the hydrogel scaffold in the surroundings of the enzyme but also a progressive leak of the enzyme by diffusion through the hydrogel toward the surrounding medium.

To quantitatively evaluate and model the kinetics of the enzymatic hydrolysis of cellulose nanofibers in the 3D printed composite hydrogels during cell culture studies, the fraction of hydrolyzed glycosidic bonds *S* = 1/*DP* − 1/*DP*_0_ was estimated for different hydrolysis times *t,* in which’s *DP*_0_ and *DP* are, respectively, the initial number average degree of polymerization and the degree of polymerization after a given hydrolysis time. The following pseudo zero-order kinetics relation (Equation (4)) has been traditionally used to model the hydrolysis of carbohydrates, such as cellulose [73,74].

(4) S=k⋅t (k: rate constant)

which is an approximation of the first-order Equation (5) [75]

(5) $$S=n^{0}\cdot \left(1-e^{-k\cdot t}\right)$$

where *n*^0^ represents the ‘accessibility’, i.e., the fraction of glycosidic bonds available for hydrolysis [76]. In heterogeneous conditions, as is the case in CNF hydrolysis, there were some issues when directly using Equation (5) to model the experimental data. Equation (4) was largely satisfied when *S* correspond to a few units, i.e., *S* << *n*^0^ or *kt* << 1. Equations (4) and (5) were effectively used for the degradation of carbohydrates in the solution, where all glycosidic bonds are available for cleavage [74,77]. Nevertheless, the carbohydrate hydrolysis kinetics in heterogeneous conditions has often been treated in the same way, even if the use of these equations is debated [74].

**Figure 10:**
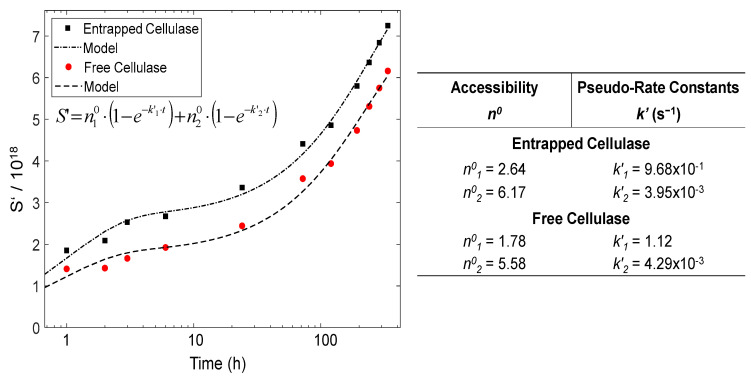
Evolution of the fraction of hydrolyzed glycosidic bonds *S*’ with the hydrolysis time during the enzymatic degradation of CNFs by the entrapped cellulase in comparison to the free enzyme. Table on the right displays the accessibility *n*^0^ and pseudo-rate constant *k*’ values obtained for the hydrolysis of CNFs by the entrapped and the free cellulase, by modeling the experimental data with the sum of parallel first-order kinetics in the regimes 1 and 2 (Equation (6)).

Figure 10 displays the time evolution of the number of hydrolyzed glycosidic bonds *S’ = S* × *DP*_0_, calculated from the obtained moles of sugar-reducing ends, as previously reported [59], for the different hydrolyzed glycosidic bonds produced during the degradation of the cellulose nanofibers for different hydrolysis times. Moreover, a pseudo-rate constant *k*’ = *k* × *DP*_0_ could be defined. As observed in Figure 10, the kinetics plots neither followed the single zero-order Equation (4) nor the first-order Equation (5) [78].

In cellulose heterogeneous hydrolysis, a global deviation was explained, invoking the initial cleavage of the so-called ‘weak links’ (w) or ‘high defects’ [61,78]. Then, distinct stages were hypothesized. A fast initial attack of the weak links, followed by a slower degradation of the remaining amorphous fraction (a) [79]; indeed, the presence of weak links was proposed to explain the differences observed in the initial rate of cellulose degradation in heterogeneous conditions [80]. As for the semi-crystalline cellulose of high *DP*_0_, which constitutes the CNFs used in the present work, its degradation should display different stages. The hydrolysis kinetics in Figure 10 shows distinct regimes. Indeed, the CNFs are composed of amorphous regions (a), with some weak links (w), and crystalline domains (c) [79]. Equation (6) could be proposed, which comprises the sum of two parallel first-order processes corresponding to the hydrolysis of w, a and c domains. We thus assume a two-regime mechanism. In regime 1, the cellulase acts on ‘weak links’ and amorphous cellulose. In regime 2, the hydrolysis is performed on the surface of crystalline domains (c) [81].

(6) $$S\prime =n_{1}^{0}\cdot \left(1-e^{-k\prime_{1}\cdot t}\right)+n_{2}^{0}\cdot \left(1-e^{-k\prime_{2}\cdot t}\right)$$

As seen in Figure 10, the way in which the cellulase is incorporated in 3D printed hydrogels, either as entrapped cellulase or as the free enzyme, has an impact on the degradation kinetics of CNFs. In the table of Figure 10, the hydrolysis pseudo-rate constants *k*’ as well the apparent ‘accessibility’ *n*^0^ values for the different regimes are summarized, which were estimated by modeling the hydrolysis data with the kinetics Equation (6). The pseudo-rate constants *k*’_1_ and *k*’_2_ were calculated, which, respectively, correspond to: (*k*’_1_) the concomitant hydrolyses of ‘weak links’ and amorphous regions; (*k*’_2_) the hydrolysis of the crystalline domains, in which slower depolymerization rate is observed, possibly due to the topological constraints inherent to the crystallites’ microstructure and the initial less accessibility of the enzyme to the intracrystalline cellulose chains [74]. Regarding accessibility, the comparison of the *n*^0^ values between the entrapped and free cellulase reveals a slightly higher apparent accessibility when using the cellulase immobilized in the nanoparticles. This apparent value may be related to the higher available amount of the enzyme, since the microstructures of cellulase-laden and virgin CNF/chitosan hydrogels were shown to be similar, in addition, with a chitosan–chitosan interface between chitosan from the nanoparticles and chitosan from the matrix of the CHI/CNF scaffold, in which the latter CHI also served as shell/coating for the cellulose nanofibrils, as demonstrated by Doench et al. [35] in our previous work about the rheological study of CHI/CNF viscous inks. Then, when comparing the accessibility values *n*^0^_1_ and *n*^0^_2_, obtained, respectively, for regime 1 (amorphous regions/weak links) and regime 2 (crystallites), in both cases, for the immobilized and for the free enzyme, the accessibility of regime 2 was surprisingly higher than that of regime 1. This could be explained by the high crystalline content of cellulose nanofibrils; after a degradation of the amorphous regions in regime 1, the chains of cellulose crystallites became accessible for the enzyme to be degraded in regime 2, even if this crystallite hydrolysis occurred at a very low reaction rate *k*’_2_ (regime 2), significantly lower than *k*’_1_ (regime 1).

## 4. Conclusions

The objective of this work was to prepare cellularized functional constructs with degradable polysaccharide scaffolds in vitro. To this end, cellulase was successfully encapsulated in chitosan–caseinate nanoparticles, which were further incorporated as nanocarriers in the process of 3D printing cellulose nanofiber-filled chitosan hydrogel scaffolds. Such encapsulation allowed a better preservation of the enzymatic activity of cellulase in comparison with the free enzyme. The encapsulation yielded a sustained and controlled degradation of nanocellulose in 3D printed CHI/CNF scaffolds, while in the short term, nanocellulose may play its mechanical role in in vitro tissue engineering. After 14 days of enzymatic hydrolysis, the encapsulated enzyme had a relative activity of approximately 65% in the degradation of cellulose nanofibers constituting 3D printed CHI/CNF hydrogel constructs. Through the fluorescence imaging of hydrogel filaments containing the encapsulated FTIC-stained cellulase enzyme at different incubation times, the presence of cellulase in the printed hydrogels was highlighted, as well as the slow and sustained release of the cellulase from chitosan–caseinate nanoparticles through the hydrogel matrix and then to the incubation medium in which the scaffolds were immersed. Subsequently, fibroblast cells were cultured on these scaffolds containing cellulase. We obtained cell survival and proliferation in the printed scaffolds after 1 and 3 days of incubation, occurring simultaneously with the degradation process of the cellulose nanofibers in the scaffolds. This investigation made it possible to demonstrate not only the biocompatibility of chitosan and cellulose nanofibers but also to propose a method for the in vitro and in vivo biodegradation of cellulose nanofibers when they are used as nanomaterials to reinforce composites for tissue engineering applications. These biodegradation studies performed in situ during cell culture are promising for the application of cellulose nanofiber-reinforced functional composites as implants with the desired controlled degradability in vivo for the regeneration and replacement of mechanically demanding tissues. These studies constitute an essential approach to predict the evolution of properties of tissue-engineered scaffolds in dynamic mimicking environments.

## 5. Patents

Osorio-Madrazo, A.; David, L.; Montembault, A.; Viguier, E.; Cachon, T. Hydrogel Composites Comprising Chitosan and Cellulose Nanofibers. International Patent Application No. WO 2019/175279 A1, 19 September 2019; US Patent App. 16/980,383, 12 February 2021.

## Data Availability

Not applicable.
